# Anti-Obesity Effect of Dyglomera^®^ Is Associated with Activation of the AMPK Signaling Pathway in 3T3-L1 Adipocytes and Mice with High-Fat Diet-Induced Obesity

**DOI:** 10.3390/molecules27103288

**Published:** 2022-05-20

**Authors:** Hae-Lim Kim, Sung-Kwon Lee, Da-Eun Min, Bong-Keun Choi, Dong-Ryung Lee

**Affiliations:** Research Institute, NUON Co., Ltd., Jungwon-gu, Seongnam 13201, Gyunggi, Korea; ics1357@naver.com (H.-L.K.); sklee@nuon.kr (S.-K.L.); demin@nuon.kr (D.-E.M.); cbcbcbk@nuon.kr (B.-K.C.)

**Keywords:** *Dichrostachys glomerata* extract, Dyglomera^®^, anti-obesity, adipogenesis, lipogenesis, lipolysis, 3T3-L1 adipocytes, high-fat diet-induced obesity

## Abstract

Dyglomera^®^ is an aqueous ethanol extract of the fruit pods of *Dichrostachys glomerata*, a Cameroonian spice. Several studies have shown its anti-diabetic and anti-obesity effects. However, the underlying mechanisms for such effects remain unclear. Thus, the objective of this study was to investigate the anti-obesity effect of Dyglomera^®^ and its underlying mechanisms in mice with high-fat diet-induced obesity and 3T3-L1 adipocytes. Our results revealed that Dyglomera^®^ inhibited adipogenesis and lipogenesis by regulating AMPK phosphorylation in white adipose tissues (WATs) and 3T3-L1 adipocytes and promoted lipolysis by increasing the expression of lipolysis-related proteins. These results suggest that Dyglomera^®^ can be used as an effective dietary supplement for treating obesity due to its modulating effect on adipogenesis/lipogenesis and lipolysis.

## 1. Introduction

Obesity is a disease associated with metabolic disorders such as nonalcoholic fatty liver disease (NAFLD), insulin resistance, hyperlipidemia, and type 2 diabetes [1]. Obesity is a complex and multifactorial chronic disease and is a serious health problem in many countries. The incidence of obesity is multifactorial. Dysregulation of lipid and glucose metabolism is considered to be an important factor [2,3]. Obesity is caused by excessive energy intake causing lipid synthesis (lipogenesis) and differentiation of pre-adipocytes into mature adipocytes (adipogenesis) in the adipose tissue [4,5]. White adipose tissue (WAT) can store excess energy as triglycerides (TG) and mobilize energy in the form of free fatty acids (FFA). High plasma lipid levels can also cause FFA not to be efficiently used by adipocytes for storage, resulting in the hepatic accumulation of lipids [6]. Therefore, lowering plasma FFA levels could be potential strategies for metabolic syndrome management. Moreover, various treatments for improving obesity include exercise therapy, diet therapy, drug therapy, and surgery. Although treatment with chemically synthesized anti-obesity drugs has high efficacy, it causes many side effects. For this reason, there is a growing interest in the use of natural plant materials that are safe and that have a low risk of side effects.

*Dichrostachys glomerata* is a plant of western Cameroon. Its fruits and seeds are edible. The fruit is a dried pod commonly used as a spice in "*Nah poh*", a traditional Cameroonian soup [7]. *D. glomerata* have been found to contain various components such as flavonoids, phenolic compounds, alkaloids, tannins, saponins, and terpenoids [8]. Recent studies have shown that *D. glomerata* has antioxidant activity [9]. Other studies have shown that *D. glomerata* has antioxidant activity in vitro and in vivo, along with low-density lipoproteins’ (LDL) antioxidant properties. *D. glomerata* has been shown to be able to lower fasting serum glucose levels and glycated hemoglobin in laboratory diabetic rats [10,11]. In addition, *D. glomerata* has been reported to improve oxidative stress in obese and type 2 diabetes patients [12,13]. Dyglomera^®^ is a standardized powder prepared by extracting *D. glomerata* fruit pods with aqueous ethanol followed by concentration and drying. Dyglomera^®^ also has anti-inflammatory and fat regulation activity in obese patients with metabolic syndrome [14,15]. Also, Dyglomera^®^ has been reported to be safe in a subchronic study in rats and genotoxicity tests [16].

However, there are few reports on the effects of the Dyglomera^®^ on obesity, underlying mechanisms, and related metabolic disorders. Therefore, the aims of the current study were to investigate the anti-obesity effects of Dyglomera^®^ and possible underlying mechanisms on 3T3-L1 adipocytes and high-fat diet induced obese mice.

## 2. Results

### 2.1. Composition of Dyglomera^®^

**Figure 1:**
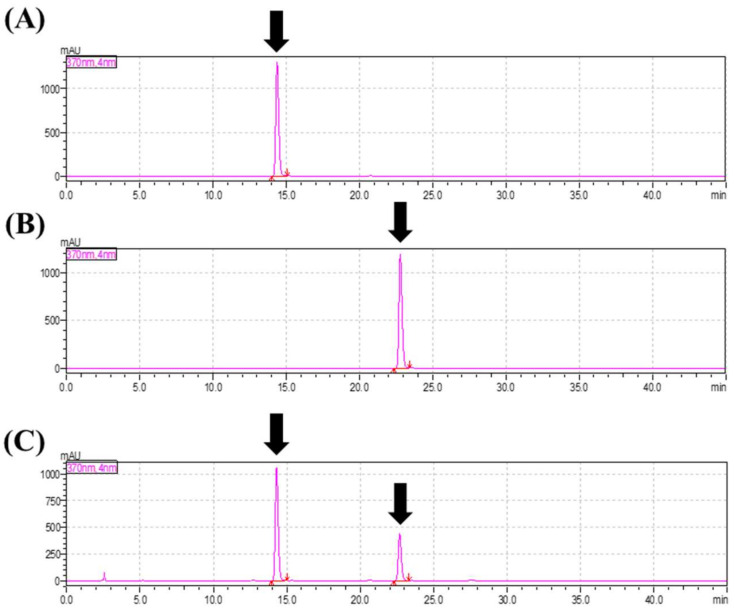
High-performance liquid chromatography (HPLC) chromatogram of myricetin, luteolin, and Dyglomera^®^. HPLC chromatogram of: (**A**) myricetin (standard); (**B**) luteolin (standard); and (**C**) Dyglomera^®^.

The marker compounds in Dyglomera^®^ were confirmed by high-performance liquid chromatography (HPLC) analysis. Quantitative analysis of Dyglomera^®^ revealed that Myricetin and Luteolin content was determined at approximately 1.6% and 1.0%, respectively (Figure 1), and the optimized Dyglomera^®^ was used for the following experiments.

### 2.2. Effects of Dyglomera^®^ on Body Weight and Food Intake in Mice with High-fat Diet-induced Obesity

**Figure 2:**
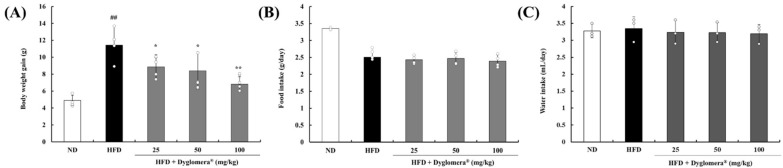
Effects of Dyglomera^®^ on body weight and food intake in mice with high-fat diet-induced obesity. (**A**) body weight, (**B**) food intake, and (**C**) water intake. ## *p* < 0.01 between the normal diet (ND) group and the high-fat diet (HFD) group. * *p* < 0.05 and ** *p* < 0.01 between HFD group and Dyglomera^®^ treated group. Data are presented as mean ± standard deviation (*n* = 5/group).

We investigated the effect of Dyglomera^®^ on obesity in high-fat diet (HFD)-induced obesity in mice. As shown in Figure 2A, the body weight gain of the HFD group was significantly increased (11.42 ± 2.31 g) when compared with the normal-fat diet (ND) group after seven weeks of feeding. In contrast, the administration of Dyglomera^®^ 25, 50, and 100 mg/kg resulted in a significant decrease in body weight gain: 8.87 ± 1.43 g (*p* < 0.05), 8.40 ± 2.18 g (*p* < 0.05), and 6.81 ± 0.88 g (*p* < 0.01), respectively (Figure 2A). On the other hand, food intake and water intake did not display significant group wise differences (Figure 2B,C).

### 2.3. Effects of Dyglomera^®^ on Plasma Biochemical Levels in Mice with High-Fat Diet-Induced Obesity

Plasma biochemical parameters such as glucose, total-cholesterol (T-CHO), triglyceride (TG), free fatty acid (FFA), low-density lipoprotein cholesterol (LDL-c), high-density lipoprotein cholesterol (HDL-c), HDL-c/LDL-c ratio, alanine transaminase (ALT), aspartate transaminase (AST), adiponectin, leptin, and blood urea nitrogen (BUN) were investigated. As shown in Table 1, serum adiponectin level and HDL-c/LDL-c ratio were decreased (*p* < 0.01), whereas serum glucose, T-CHO, FFA, LDL-c, ALT, AST, leptin, and BUN levels were increased (*p* < 0.05) in the HFD group. However, glucose, T-CHO, ALT, AST, and leptin levels in groups administered with Dyglomera^®^ 25 (*p* < 0.05), 50 (*p* < 0.05), and 100 (*p* < 0.01) were significantly decreased as compared to those in the HFD group. TG, FFA, and BUN levels were significantly decreased (*p* < 0.01) in groups treated with Dyglomera^®^. LDL-c levels I groups administered with Dyglomera^®^ were also decreased (*p* < 0.05), whereas HDL-c showed a tendency to increase in the group administrated with Dyglomera^®^. HDL-c/LDL-c ratio and adiponectin levels were increased (*p* < 0.01) in the group administrated with Dyglomera^®^.

### 2.4. Effects of Dyglomera^®^ on WATs in Mice with High-Fat Diet-Induced Obesity

**Figure 3:**
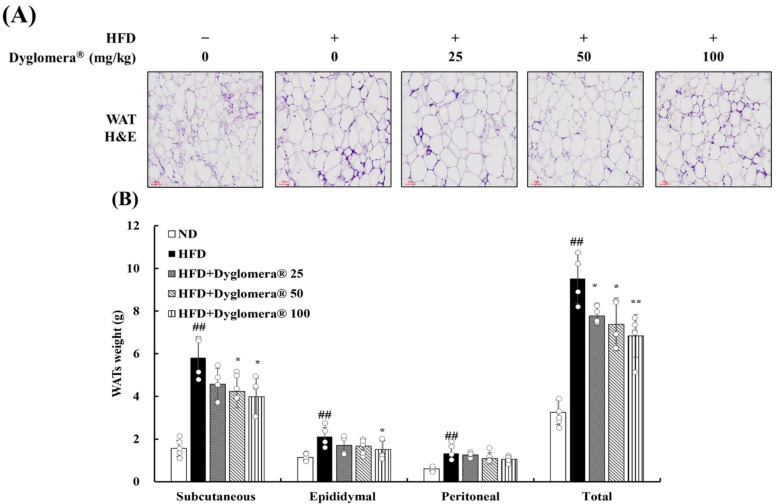
Effects of Dyglomera^®^ on WATs in mice with high-fat diet-induced obesity. (**A**) The WATs were stained with hematoxylin and eosin (H&E) and observed under a microscope. (**B**) The WATs were weighed. Scale bar = 100 μm. ## *p* < 0.01 between normal diet (ND) group and high fat diet (HFD) group. * *p* < 0.05 and ** *p* < 0.01 between HFD group and Dyglomera^®^ treated group.

H&E staining results of white adipose tissues (WATs) showed that the size of adipocytes decreased in the groups treated with Dyglomera^®^ (Figure 3A).

Moreover, subcutaneous adipose tissue, epididymal adipose tissue, peritoneal adipose tissue, and total WATs weight were also measured to determine the effect of Dyglomera^®^ on organ weights. The weight of WATs was markedly increased (*p* < 0.01) in the HFD group compared to that in the ND group. Subcutaneous adipose tissue weight decreased (*p* < 0.05) in the Dyglomera^®^ 50 and 100 groups. Epididymal adipose tissue weight decreased (*p* < 0.05) in the Dyglomera^®^ 100 group. However, peritoneal adipose tissue only showed a tendency to decrease without showing statistical significance. Total WATs weight was significantly decreased by administration of Dyglomera^®^ in a dose-dependent manner (in Dyglomera^®^ 25 and 50 groups, *p* < 0.05: in 100 group, *p* < 0.01) (Figure 3B).

### 2.5. Effects of Dyglomera^®^ on Expression of Adipogenesis and Lipogenesis Related Proteins in Epididymal WATs

**Figure 4:**
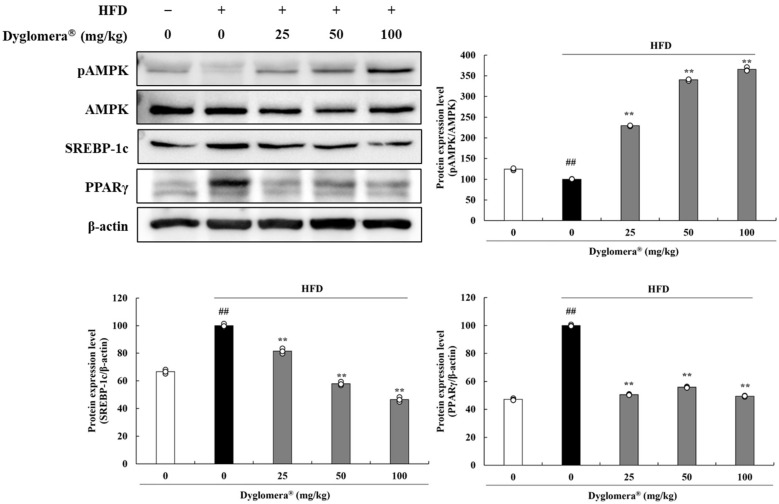
Effects of Dyglomera^®^ on expression of adipogenesis and lipogenesis related proteins in epididymal WATs. ## *p* < 0.01 between normal diet (ND) group and high fat diet (HFD) group. ** *p* < 0.01 between HFD group and Dyglomera^®^ treated group. Data are presented as mean ± standard deviation of three independent experiments.

We also investigated phospho-AMP-activated protein kinase (pAMPK), sterol regulatory element-binding protein-1c (SREBP-1c), and peroxisome proliferator-activated receptor γ (PPARγ) expression in epididymal WATs. As a result, phosphorylation of AMPK was decreased in the HFD administrated group (24.63%, *p* < 0.01) compared to the ND group, whereas 25, 50 and 100 mg/kg Dyglomera^®^ administrated groups dose dependently increased by 129.44%, 240.31%, and 265.58% (*p* < 0.01). In epididymal WATs of the HFD group, SREBP-1c, and PPARγ protein levels were significantly reduced compared to those in the ND group. SERBP-1c protein levels were significantly suppressed by 18.44%, 42.18%, and 53.46% in the Dyglomera^®^ 25, 50 and 100 mg/kg administrated groups. Also, PPARγ protein levels were significantly suppressed by 49.48%, 44.14%, and 50.54% in the Dyglomera^®^ 25, 50 and 100 mg/kg administrated groups (Figure 4).

### 2.6. Effects of Dyglomera^®^ on Lipolysis Related Proteins in Epididymal WATs

**Figure 5:**
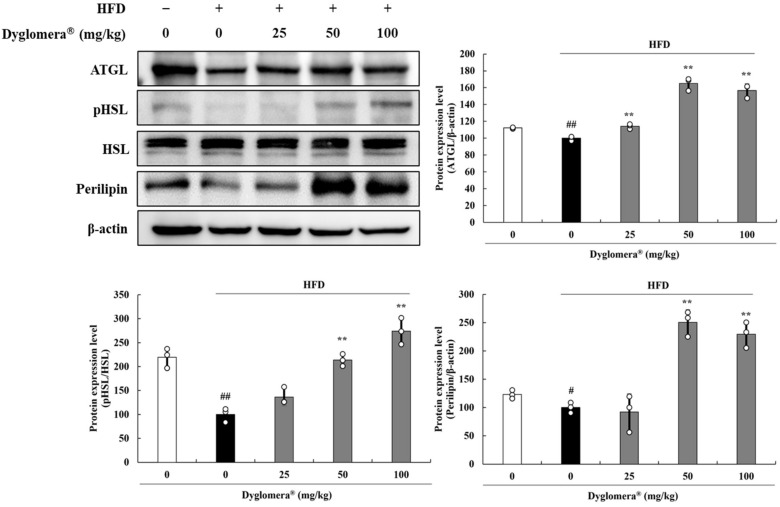
Effects of Dyglomera^®^ on lipolysis-related proteins in epididymal WATs. # *p* < 0.05, ## *p* < 0.01 between normal diet (ND) group and high fat diet (HFD) group. ** *p* < 0.01 between HFD group and Dyglomera^®^ treated group. Data are presented as mean ± standard deviation of three independent experiments.

In epididymal WATs of the HFD group, adipose triglyceride lipase (ATGL), phospho-hormone sensitive lipase (pHSL), and perilipin protein levels were significantly reduced compared to those in the ND group. ATGL was increased by 14.3%, 65.0%, and 56.7% in the Dyglomera^®^ 25, 50, and 100 groups, respectively (*p* < 0.01). pHSL/HSL protein levels were increased by 85.4% and 329.8% in the Dyglomera^®^ 50 and 100 groups, respectively (*p* < 0.01). At the same concentration, perilipin protein was also increased by 106.8% and 92.6%, respectively (*p* < 0.01) (Figure 5).

### 2.7. Cytotoxic Effects of Dyglomera^®^ on Viability of 3T3-L1 Preadipocytes

**Figure 6:**
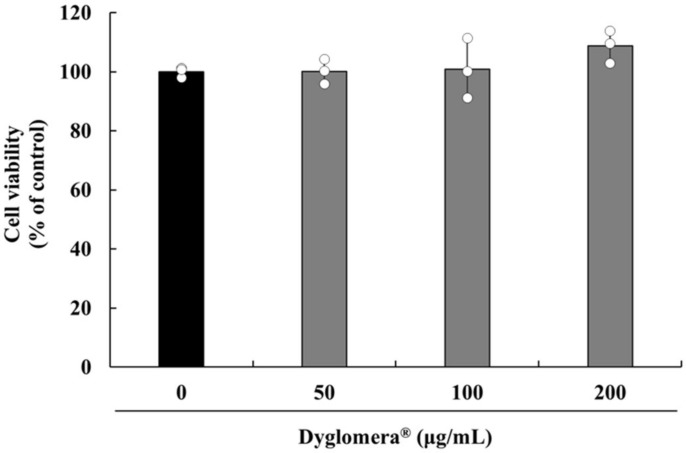
Cytotoxic Effects of Dyglomera^®^ on viability of 3T3-L1 preadipocytes. There were no significant differences in cell viability between Dyglomera^®^ 50, 100, or 200 μg/mL treated groups and those not treated with Dyglomera^®^. Data are presented as mean ± standard deviation of triplicate experiments.

The cytotoxic effect of Dyglomera^®^ on 3T3-L1 preadipocytes was evaluated using the 3-(4,5-dimethylthiazol-2-yl)-2,5- diphenyl tetrazolium bromide (MTT) assay. Dyglomera^®^ did not exhibit cytotoxicity at any concentrations investigated (50, 100, or 200 μg/mL, Figure 6).

### 2.8. Effects of Dyglomera^®^ on Lipid Accumulation in 3T3-L1 Preadipocytes

**Figure 7:**
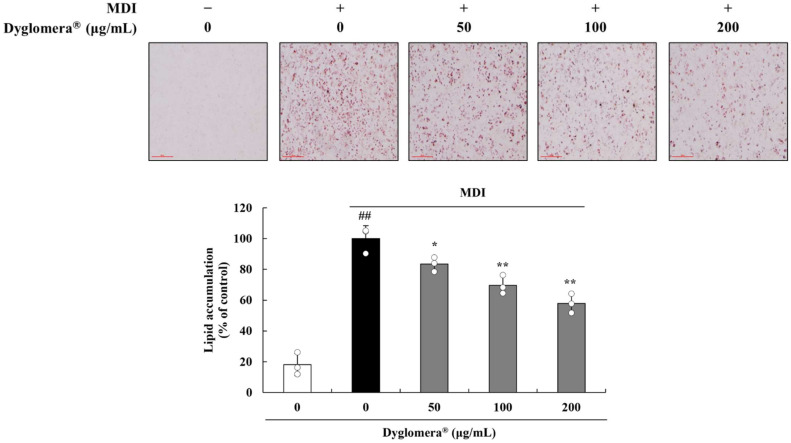
Effects of Dyglomera^®^ on lipid accumulation in 3T3-L1 preadipocytes. 3T3-L1 preadipocytes were differentiated to mature adipocytes with differentiation media supplemented with or without Dyglomera^®^ extract. Lipid accumulation was quantified by Oil-red O staining and absorbance at 520 nm. Control group (“0 μg/mL”), only dimethyl sulfoxide (DMSO) treated no differentiation control; MDI, 3-isobutyl-1-methylxanthine, dexamethasone, and insulin treated differentiation group. Scale bar = 500 μm. ## *p* < 0.01 vs. control group. * *p* < 0.05, ** *p* < 0.01 vs. MDI control group. Data are presented as mean ± standard deviation of three independent experiments.

To investigate the effect of Dyglomera^®^ on preadipocyte differentiation, 3T3-L1 preadipocytes were treated with MDI (3-isobutyl-1-methylxanthine, dexamethasone, and insulin) differentiation medium (DMEM, 10% fetal bovine serum (FBS), 0.5 mM IBMX, 5 µg/mL insulin, and 1 µM dexamethasone) with or without Dyglomera^®^ (50, 100, or 200 μg/mL). Lipid droplets were stained with Oil-red O staining and lipid concentrations were quantified. Results showed that Dyglomera^®^ decreased the accumulation of lipid droplets in a dose-dependent manner. Lipid accumulation was reduced by 57.9% (*p* < 0.05), 64.5% (*p* < 0.01), and 83.4% (*p* < 0.01), respectively, in Dyglomera^®^-treated groups at 50, 100, and 200 µg/mL concentrations (Figure 7).

### 2.9. Effects of Dyglomera^®^ on Expression of Adipogenesis and Lipogenesis Related Proteins in 3T3-L1 Adipocytes

**Figure 8:**
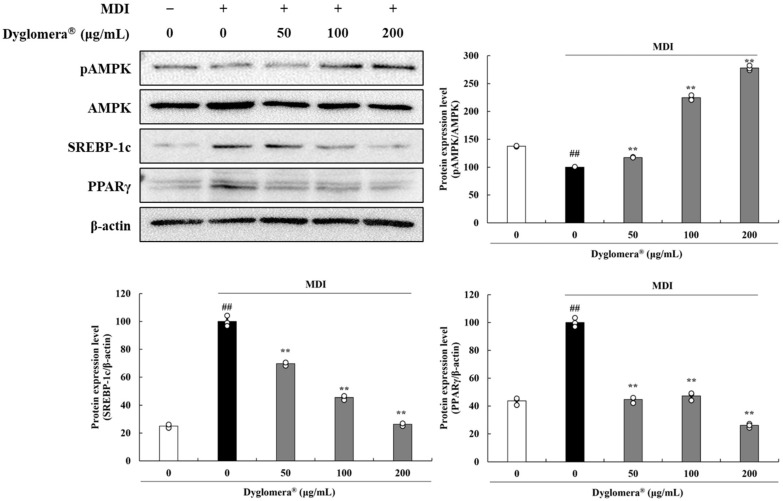
Effects of Dyglomera^®^ on expression of adipogenesis and lipogenesis-related proteins in 3T3-L1 adipocytes. Control group (“0 μg/mL”), only dimethyl sulfoxide (DMSO) treated no differentiation control; MDI, 3-isobutyl-1-methylxanthine, dexamethasone, and insulin treated differentiation group. ## *p* < 0.01 vs. control group. ** *p* < 0.01 vs. MDI control group. Data are presented as mean ± standard deviation of three independent experiments.

To investigate the effects of Dyglomera^®^ on adipogenesis and lipogenesis, proteins were extracted from 3T3-L1 adipocytes and epididymal WATs, respectively. AMPK is a major regulator of cellular energy homeostasis. It regulates carbohydrate and fat metabolism in order to maintain cellular energy balance. SREBP-1c is a target protein directly regulated by AMPK. It has been identified as one of the transcription factors involved in adipogenesis/lipogenesis. SREBP-1c is also known to induce the expression of PPARγ. To determine the association between Dyglomera^®^ and the activation of AMPK, we determined whether Dyglomera^®^ could induce AMPK phosphorylation during 3T3-L1 differentiation. It was found that the pAMPK/AMPK level was markedly increased in Dyglomera^®^ 50, 100, and 200 μg/mL treated 3T3-L1 cells by 17.09%, 124.42%, and 177.79%, respectively (*p* < 0.01). Furthermore, levels of SREBP-1c and PPARγ were significantly suppressed in 50, 100, and 200 μg/mL Dyglomera^®^ treated 3T3-L1 cells (*p* < 0.01) (Figure 8).

### 2.10. Effects of Dyglomera^®^ on Lipolysis Related Proteins in 3T3-L1 Adipocytes

**Figure 9:**
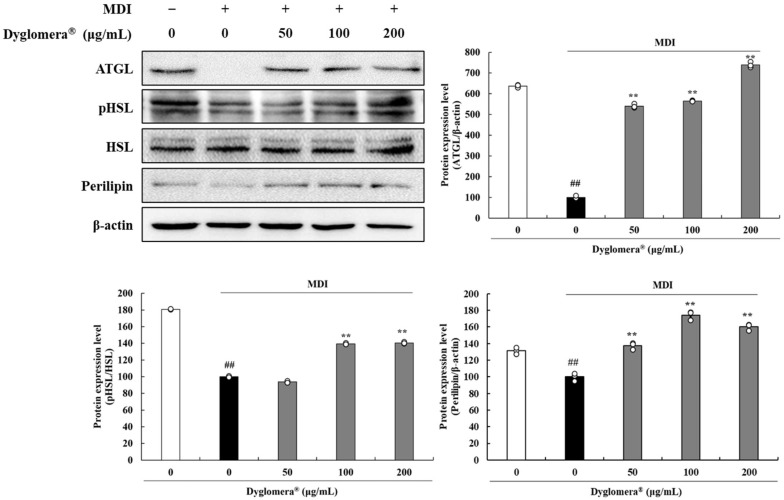
Effects of Dyglomera^®^ on lipolysis-related proteins in 3T3-L1 adipocytes. Control group (“0 μg/mL”), only dimethyl sulfoxide (DMSO) treated no differentiation control; MDI, 3-isobutyl-1-methylxanthine, dexamethasone, and insulin treated differentiation group. ## *p* < 0.01 vs. control group. ** *p* < 0.01 vs. MDI control group. Data are presented as mean ± standard deviation of three independent experiments.

In this study, lipolysis-related proteins were evaluated in 3T3-L1 cells and epididymal WATs. As shown in Figure 9, ATGL, pHSL, and perilipin protein expression levels were decreased in 3T3-L1 cells, whereas treatment with Dyglomera^®^ 50, 100, and 200 μg/mL increased ATGL expression by 440.6%, 465.3%, and 639.1%, respectively. Dyglomera^®^ at 100 and 200 μg/mL increased pHSL/HSL by 39.3% and 40.4%, respectively. Dyglomera^®^ at 50, 100, and 200 μg/mL increased perilipin by 37.4%, 74.2%, and 59.9%, respectively (*p* < 0.01).

## 3. Discussion

Obesity is a multifactorial and chronic disease caused by the excessive accumulation of lipids. Obesity represents a serious health problem worldwide as it can increase the risk of metabolic diseases. These days, many herbal plants have been studied for anti-obese properties. Among them, the fruit of *Dichrostachys glomerata* has received special attention as a safe and lipid-lowering plant in Cameroon [15,16].

To investigate the anti-obesity effects and underlying mechanisms of Dyglomera^®^, an HFD-induced obese mouse model and 3T3-L1 cells were used in this study. They are the most widely used models for obesity studies [17,18]. 3T3-L1 adipocytes are an effective cell model for studying mechanisms of adipocyte differentiation and lipid accumulation [19,20]. 

It is well-known that feeding HFD can readily induce obesity such as body weight gain and weight gain of WATs [21]. Administration of Dyglomera^®^ was shown to lower body weight and total weight WATs markedly in HFD-administrated mice, whereas Dyglomera^®^ treatment did not affect the food intake and water intake of mice with HFD-induced obesity. These results indicate that Dyglomera^®^ has an anti-obesity effect.

Dyglomera^®^ supplementation significantly reduced plasma glucose, T-CHO, TG, FFA, and LDL-c levels while increasing the plasma HDL-c/LDL-c ratio in HFD-fed mice. These results suggest that Dyglomera^®^ can reduce hyperglycemia and hyperlipidemia caused by HFD. It is well-known that serum levels of AST and ALT, the major enzymes present in hepatocytes, are increased after hepatocellular injury [22]. Excessive intake of high fat can induce oxidative stress, which can lead to liver dysfunction and fatty degeneration. ALT is a sensitive biomarker of NAFLD [23]. Dyglomera^®^ administration significantly reduced serum AST and ALT levels increased by HFD. The serum levels of BUN were determined to evaluate kidney function [24]. The increase in serum BUN level was observed in HFD-treated mice. However, Dyglomera^®^ treatment significantly reduced serum BUN levels in HFD-induced obese mice. The findings showed administration of Dyglomera^®^ to HFD-induced mice has remarkably reduced plasma BUN levels, suggesting that Dyglomera^®^ may prevent renal injury resulting from long-term high-fat diet intake. In obesity, the accumulation of fat affects adipokines including leptin and adiponectin in adipose tissues [25]. Adiponectin is known to increases insulin sensitivity, glucose uptake, and fatty acid oxidation effects of hormone-stimulated lipolysis [26]. Leptin levels were significantly increased in obese mice. These elevated leptin levels suggest that most obese individuals develop leptin resistance, which prevents them from suppressing eating [27]. However, Dyglomera^®^ treatment decreased leptin levels and conversely increased adiponectin levels.

We also performed an MTT assay to confirm the cytotoxicity of Dyglomera^®^. As a result, no significant toxicity was observed for Dyglomera^®^ at concentration of 50, 100, or 200 μg/mL. As a result of Oil-red O staining, Dyglomera^®^ treatment in 3T3-L1 decreased the accumulation of lipid droplets in a dose-dependent manner. To examine the underlying mechanism, we examined expression levels of adipogenesis/lipogenesis and lipolysis related proteins in WATs of mice with HFD-induced obesity and 3T3-L1 cells. AMPK plays a major role in regulating the lipid synthesis pathway and energy metabolism in adipocytes [28]. When AMPK is phosphorylated, the catabolic pathway is turned on and the ATP-consuming anabolic pathway is simultaneously turned off [29]. Activation of AMPK can suppress the expression of SREBP-1c and PPARγ in adipogenesis/lipogenesis [30]. AMPK can also stimulate mitochondrial fatty acid oxidation and lipolysis [31]. Lipolysis, the catabolic pathway of the fatty acid cycle, is also crucial to balanced fat metabolism. Enhanced lipolysis contributes to the release of fatty acids and a decrease in fat deposition [32]. Expression of pHSL, ATGL, and perilipin are key factors regulating the lipolysis pathway in adipose tissues by cleaving ester bonds to degrade triglycerides [33,34,35]. In our study, phosphorylation of AMPK was significantly increased by Dyglomera^®^ treatment in WATs and 3T3-L1 cells. Expression levels of adipogenesis/lipogenesis related proteins such as SREBP-1c and PPARγ were suppressed by Dyglomera^®^ treatment in WATs and 3T3-L1 cells. Expression levels of lipolysis-related proteins such as pHSL, ATGL, and perilipin were increased by Dyglomera^®^ treatment in WATs and 3T3-L1 cells.

**Figure 10:**
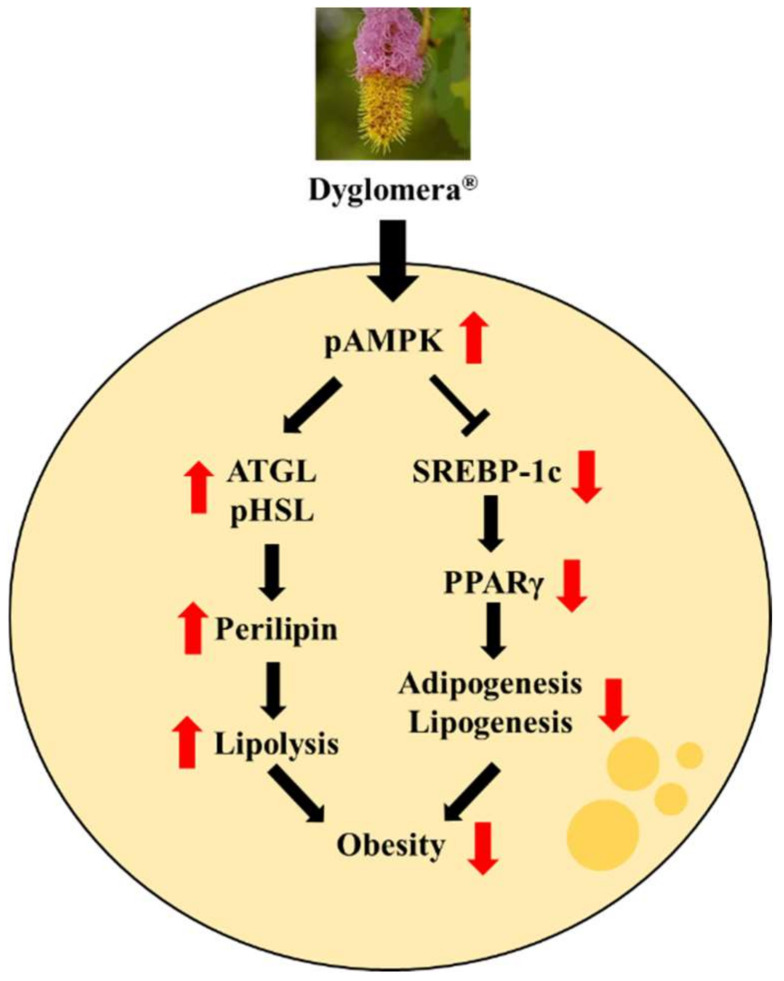
The proposed anti-obesity effects mechanism of Dyglomera^®^.

Taken together, Dyglomera^®^ could inhibit adipogenesis/lipogenesis by regulating AMPK phosphorylation in WATs and 3T3-L1 cells. In addition, Dyglomera^®^ could promote lipolysis by increasing the expression of lipolysis-related proteins (Figure 10). Moreover, Dyglomera^®^ lowered serum lipid and glucose levels.

These results suggested that Dyglomera^®^ can be used as an effective functional food for treating obesity. In addition, further studies such as a comprehensive laboratory animal monitoring system (CLAMS) [36] are needed to evaluate the effects of Dyglomera^®^ in activities, even circadian rhythms. Also, it is suggested that further mechanism studies through treatment with AMPK inhibitors such as compound C are needed to elucidate the AMPK-related anti-obesity biochemical mechanism of Dyglomera^®^. 

## 4. Materials and Methods

### 4.1. Chemicals and Reagents

Dulbecco’s modified Eagle’s medium (DMEM), bovine calf serum (BCS), and penicillin/streptomycin were purchased from Gibco BRL (Grand Island, NY, USA). Fetal bovine serum (FBS) was obtained from ATLAS Biologicals (Fort Collins, CO, USA). Insulin, 3-isobutylmethylxanthine, and dexamethasone were obtained from Wako Pure Chemical Industries Ltd. (Osaka, Japan). Oil-red O solution was purchased from Sigma-Aldrich (St. Louis, MO, USA). Isopropanol was obtained from Daejung Chemical (Seoul, Korea). Primary antibodies against phospho-AMP-activated protein kinase (pAMPK), AMPK, adipose triglyceride lipase (ATGL), phospho-hormone sensitive lipase (pHSL), HSL, perilipin, and β-actin were purchased from Cell Signaling Technology (Danvers, MA, USA). Peroxisome proliferator-activated receptor γ (PPARγ), and sterol regulatory element-binding protein-1c (SREBP-1c) were obtained from Santa Cruz Biotechnology (Santa Cruz, CA, USA). Horseradish peroxidase (HRP)-linked anti-rabbit IgG and HRP-linked anti-mouse IgG were purchased from GenDEPOT (Barker, TX, USA).

### 4.2. Sample Preparation

Dyglomera^®^, an aqueous ethanol extract of *Dichrostachys glomerata* fruit pods (standardized to Myricetin 1.6% and Luteolin 1.0%), was supplied by Gateway Health Alliances, Inc. (Fairfield, CA, USA). The manufacturing process was as follows: *Dichrostachys glomerata* fruit pods were extracted using aqueous ethanol. The resulting solution was concentrated and dried to yield Dyglomera^®^. For in vitro studies, Dyglomera^®^ was dissolved in dimethyl sulfoxide (DMSO) at a concentration of 50, 100, or 200 mg/mL and then diluted with culture medium at concentration of 50, 100, or 200 μg/mL., respectively. Control 3T3-L1 cells were treated with culture medium containing only DMSO (final DMSO concentration 0.1%). For in vivo studies, Dyglomera^®^ was homogenized in 0.5 mL distilled water at a concentration of 25, 50, or 100 mg/kg, respectively and orally administered to mice. Moreover, the dose used in this study in mice represents a feasible dose in humans [37]. Control mice were given the same volume of distilled water instead of the test solution.

### 4.3. 3T3-L1 Preadipocytes Culture and Differentiation

3T3-L1 preadipocytes (American Type Culture Collection, Rockville, MD, USA) were cultured in DMEM with 10% bovine calf serum (BCS) and 1% at 37 °C in a humified atmosphere with 5% CO_2_. To differentiate 3T3-L1 preadipocytes into mature adipocytes, fully confluent preadipocytes were treated with MDI (3-isobutyl-1-methylxanthine, dexamethasone, and insulin) differentiation medium (DMEM, 10% fetal bovine serum (FBS), 0.5 mM IBMX, 5 µg/mL insulin, and 1 µM dexamethasone) with or without Dyglomera^®^ (defined as Day 0). After two days of incubation, the MDI differentiation medium was replaced with DMEM supplemented with 10% FBS and 5 μg/mL insulin in the presence or absence of Dyglomera^®^ (Day 2). After another two days of incubation, the medium was replaced with DMEM containing 10% FBS (Day 4). On day 6, 3T3-L1 preadipocytes were fully differentiated into mature adipocytes.

### 4.4. Oil-Red O Staining

On day six, differentiated 3T3-L1 adipocytes were washed with phosphate-buffered saline (PBS) and fixed with a 4% formalin solution (Sigma-Aldrich, St. Louis, MO, USA) for 1 h. After fixation, cells were treated with Oil-red O solution and stained for 4 h. Each well was washed several times with distilled water and photographed with an ECLIPSE Ts2 microscope (Nikon Corporation, Tokyo, Japan). Stained lipids were then eluted with isopropanol and the absorbance was measured at 520 nm using a microplate reader (Tecan, Mannedorf, Switzerland).

### 4.5. Cell Cytotoxicity Activity

A 3-(4,5-dimethylthiazol-2-yl)-2,5- diphenyl tetrazolium bromide (MTT) assay was performed to determine the cytotoxicity of Dyglomera^®^ to 3T3-L1 preadipocytes. Cells were treated with Dyglomera^®^ (50, 100 or 200 μg/mL) for 24 h. After MTT solution (5 mg/mL) was added to each well, and cells were incubated at 37 °C for 3 h. After removing the supernatant, formazan crystals were eluted with DMSO. Absorbance at 570 nm was measured with a microplate plate reader (Tecan, Mannedorf, Switzerland).

### 4.6. Animals and Diet

Animal experimental procedures were approved by the Institutional Animal Care and Use Committee (IACUC) of Gyeonggido Business & Science Accelerator (GBSA) BIO CENTER (Suwon, Korea) (Permission No.: 2020-02-0002). Male C57BL/6J mice at five weeks old were obtained from Orient Bio Inc (Seongnam, Korea). Mice were maintained with controlled conditions (temperature of 22.0 ± 3.0 °C, humidity of 50.0 ± 20.0%, and a 12 h light/dark cycle). During acclimatization for one week, mice were given free access to water and food (Teklad Certified Irradiated Global 18% Protein Rodent Diet, 2918C, Envigo RMS, Inc., Indianapolis, IN, USA).

After acclimatization, mice were fed either a normal-fat diet (ND, *n* = 7) as a normal group or high-fat diet (HFD, *n* = 28, rodent diet with 60% kcal fat (#D12492, Research diets INC)) as an HFD-induced obesity group for seven weeks. After obesity induction, mice with obesity were further divided into the following four experimental groups (*n* = 7/group) and matched by body weight: HFD group; Dyglomera^®^ 25 group (high-fat diet + Dyglomera^®^ 25 mg/kg); Dyglomera^®^ 50 group (high-fat diet + Dyglomera^®^ 50 mg/kg); and Dyglomera^®^ 100 group (high-fat diet + Dyglomera^®^ 100 mg/kg). These groups were monitored for seven weeks. Body weight and food intake were measured twice a week after group separation. At the end of the experiment period, mice were fasted for 12 h and euthanized. Blood was collected and adipose tissues were rapidly removed, rinsed with physiological saline solution, weighed, and stored at −80 °C.

### 4.7. Biochemical Analysis

The blood collected before autopsy was stabilized in a blood collection tube containing an anticoagulant (EDTA-2k). Serum was prepared after centrifugation at 13,000 rpm for 15 min at 4 °C and then stored at −80 °C until use. Glucose, total-cholesterol (T-CHO), triglyceride (TG), low-density lipoprotein cholesterol (LDL-c), high-density lipoprotein cholesterol (HDL-c), alanine transaminase (ALT), aspartate transaminase (AST), and blood urea nitrogen (BUN) levels were measured using a blood biochemical analyzer (Hitachi 7020, Tokyo, Japan). The contents of the free fatty acids (FFA) leptin and adiponectin in the blood were analyzed using an enzyme-linked immunosorbent assay (ELISA) kit (Abcam, Cambridge, UK).

### 4.8. Histological Analysis

Adipose tissues were fixed with 10% neutral formalin. Tissues were embedded in paraffin and stained with hematoxylin and eosin (H&E) stain. Stained slides were photographed under a microscope (TE2000-U; Nikon, Tokyo, Japan).

### 4.9. Protein Extraction and Western Blot Analysis

3T3-L1 adipocytes were lysed with CelLytic buffer (Sigma-Aldrich, MO, USA). The cell lysate was centrifuged at 13,000 rpm for 15 min at 4 °C. Epididymal adipose tissues were washed with cold PBS and homogenized with RIPA buffer containing 50 mM Tris-HCl pH 7.4, 150 mM NaCl, 1 mM EDTA, 1% Triton X-100, 1% sodium deoxycholate, 0.1% SDS, 1 mM PMSE, and 1% protease inhibitor. The homogenate was centrifuged at 13,000 rpm for 15 min at 4 °C. Cells and adipose tissue supernatant were collected and protein concentration was measured with Bradford assay (Bio-Rad Laboratories, Hercules, CA, USA) and were then quantified to have the same protein concentration in the same volume (20 μg/20 μL). Proteins were separated by sodium dodecyl sulfate polyacrylamide gel electrophoresis (SDS-PAGE) and transferred to an Immobilon-P membrane (Millipore, Bedford, MA, USA). The transferred membrane was blocked with 5% skim milk for 1 h and then incubated with specific primary antibodies at 4 °C overnight. Blots were washed with tris buffered saline with 0.5% Tween 20 (TBS-T) three times and then incubated with corresponding horseradish peroxidase-conjugated anti-rabbit or anti-mouse immunoglobulin G at room temperature for 1 h. The intensity of each band detected by using ECL solution (GenDEPOT, Barker, TX, USA) was measured with a LuminoGraph (Atto, Tokyo, Japan). Band images were digitized using the ImageJ program developed at the National Institutes of Health (NIH; Bethesda, MD, USA) and corrected by β-actin level. There was no significant difference between all β-actin-to-β-actin levels.

### 4.10. Statistical Analysis

Data are presented as mean ± standard deviation. Values were compared using Student’s *t*-test. One-way analysis of variance (ANOVA) was performed using the software Origin 7 (Microcal Software, Northampton, MA, USA). Values of *p* < 0.05 and *p* < 0.01 indicated statistical significance.

## Figures and Tables

**Table 1:** Effects of Dyglomera^®^ on plasma biochemical levels in mice with high-fat diet-induced obesity. Data are presented as mean ± standard deviation (*n* = 5/group). # *p* < 0.05, ## *p* < 0.01 between the normal diet (ND) group and the high-fat diet (HFD) group. * *p* < 0.05 and ** *p* < 0.01 between the HFD group and the Dyglomera^®^ treated group.

Indexes	ND	HFD	HFD		
			Dyglomera^®^ 25	Dyglomera^®^ 50	Dyglomera^®^ 100
Glucose (mg/dL)	278.31 ± 93.32	549.21 ± 199.22 ##	345.09 ± 127.80 *	335.02 ± 132.27 *	305.61 ± 43.53 **
T-CHO (mg/dL)	130.77 ± 9.06	209.04 ± 29.60 ##	162.89 ± 38.35 *	168.04 ± 19.71 *	144.31 ± 35.13 **
TG (mg/dL)	50.89 ± 9.17	46.79 ± 14.06	27.95 ± 5.81 **	28.15 ± 5.87 **	25.65 ± 2.72 **
FFA (nmol/μL)	0.88 ± 0.03	1.48 ± 0.00 ##	1.14 ± 0.01 **	1.02 ± 0.02 **	0.87 ± 0.05 **
LDL-c (mg/dL)	4.64 ± 0.55	15.35 ± 3.52 ##	10.63 ± 3.06 *	10.04 ± 2.97 *	6.01 ± 1.78 *
HDL-c (mg/dL)	95.05 ± 15.01	82.19 ± 18.07	98.22 ± 8.11	98.76 ± 5.50	99.48 ± 9.64
HDL-c/LDL-c	20.47 ± 1.63	5.39 ± 0.58 ##	11.03 ± 2.27 **	11.11 ± 2.42 **	14.83 ± 1.97 **
ALT (U/L)	27.02 ± 2.22	296.38 ± 116.48 ##	164.97 ± 87.61 *	166.09 ± 78.63 *	71.09 ± 36.92 **
AST (U/L)	102.68 ± 13.11	262.21 ± 102.73 ##	157.32 ± 19.98 *	155.13 ± 55.68 *	132.33 ± 25.99 **
Adiponectin (ng/mL)	42.27 ± 0.77	33.01 ± 0.95 ##	35.85 ± 1.78 **	38.34 ± 0.28 **	38.84 ± 1.28 **
Leptin (ng/mL)	7.52 ± 1.21	65.38 ± 6.04 ##	55.54 ± 1.67 *	50.77 ± 9.02 *	44.31 ± 9.03 **
BUN (mg/dL)	28.17 ± 2.90	32.24 ± 2.72 #	23.46 ± 4.12 **	23.31 ± 2.84 **	21.02 ± 5.17 **

## Data Availability

Not applicable.

## Sample Availability

Samples of the compounds are not available from the authors.
